# Mr. John Macphail

**Published:** 1910-06-18

**Authors:** 


Mr. John Macphail has died in London at the age of fifty-four. He studied at Glasgow, where he took the M.B. and C.M. in 1886. His hospital appointments included those of hon. surgeon to the Barnsley Brevett Hospital, Yorkshire, and house physician to the City of Glasgow Fever Hospital, besides several consulting posts to various assurance and other companies.

